# Can prenatal diagnosis of parachute mitral valve be achieved? A case report of fetal parachute mitral valve

**DOI:** 10.1186/s12947-022-00288-z

**Published:** 2022-07-08

**Authors:** Xiaohui Dai, Jiao Chen, Hanmin Liu, Lin Wu, Fumin Zhao

**Affiliations:** 1 Department of Ultrasonic Medicine, West China Second University Hospital of Sichuan University, Chengdu, 610041 Sichuan China; 2 Key Laboratory of Birth Defects and Related Diseases of Women and Children (Sichuan University), Ministry of Education, Chengdu, 610041 Sichuan China; 3 Department of Pediatrics, West China Second University Hospital of Sichuan University, Chengdu, 610041 Sichuan China; 4 Department of Obstetrics, West China Second University Hospital of Sichuan University, Chengdu, 610041 Sichuan China; 5 Department of Radiology, West China Second University Hospital of Sichuan University, Chengdu, 610041 Sichuan China

**Keywords:** Parachute mitral valve, Congenital mitral stenosis, Parachute-like asymmetric mitral valve, Prenatal diagnosis, Echocardiography

## Abstract

Parachute mitral valve (PMV) is a common form of congenital mitral stenosis and is difficult to diagnose prenatally. This report describes a fetal case of PMV with coarctation of the aorta that was diagnosed at 25 weeks’ gestation by echocardiography and confirmed at autopsy. We describe the ultrasonographic features in this case and present a useful sign for making a prenatal diagnosis of PMV.

**Supplementary Information:**

The online version contains supplementary material available at 10.1186/s12947-022-00288-z.

## Background

Parachute mitral valve (PMV) is a congenital anomaly of the papillary muscles (PMs) and frequently leads to mitral stenosis (MS). Fetal MS has often been reported, but accurate assessment of the anatomy of the PMs is difficult in the fetus [1]. Prenatal diagnosis of PMV is challenging. In this report, we describe a case of fetal PMV that was diagnosed by echocardiography and confirmed to be parachute-like asymmetric mitral valve (PLAMV) at autopsy.

## Case presentation

**Fig. 1:**
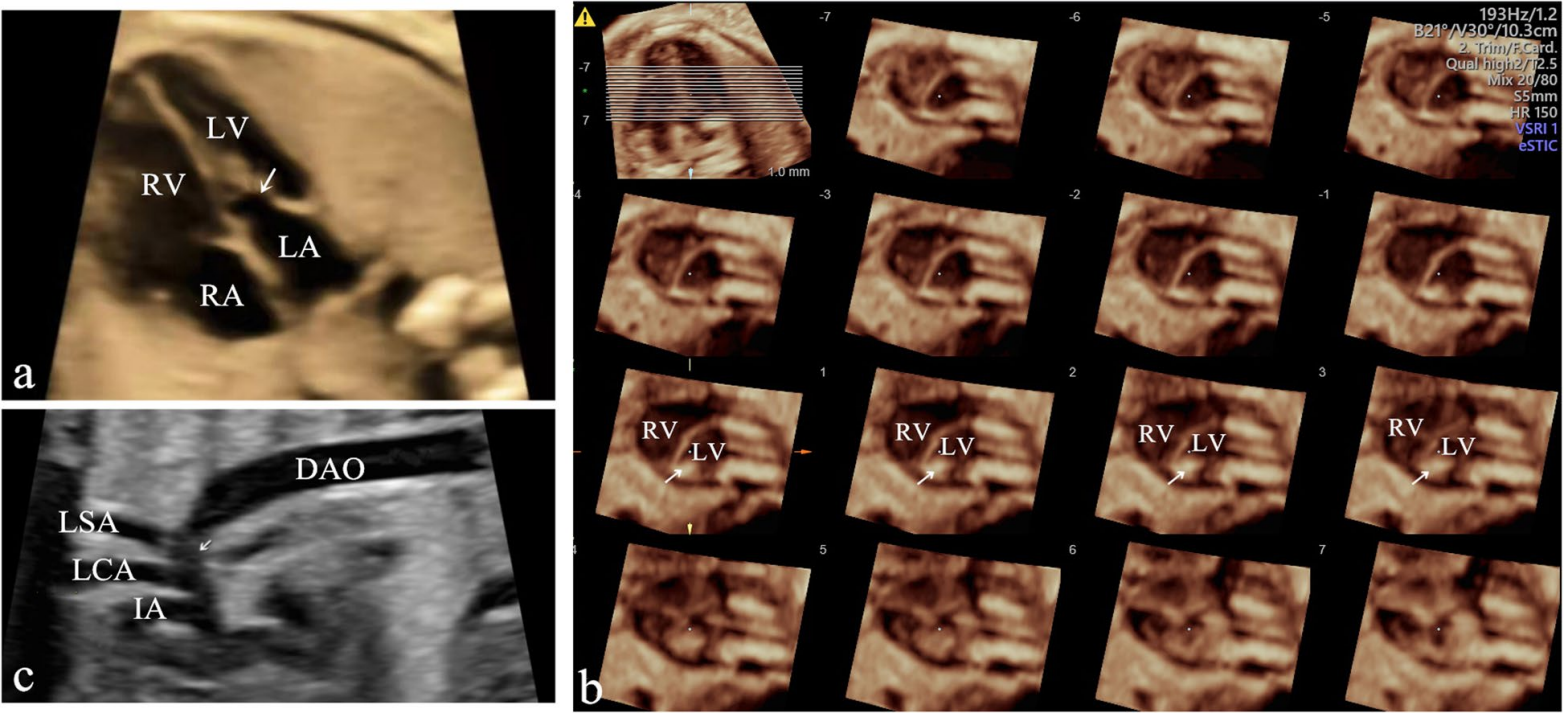
Fetal echocardiographic images. **a** The four-chamber view showing the shape and limited opening of the mitral valve, which has thickened chordae that converge into a single papillary muscle (PM) in diastole, similar to “Ω” shape (arrow). **b** Four-dimensional tomographic ultrasound images with short-axis views of the left ventricle at different levels that detected the posteromedial PM (arrow) without anterolateral PM. **c** Long-axis view of the aortic arch showing marked narrowing of the arch and isthmus (arrow). DAO, descending aorta; IA, innominate artery; LA, left atrium; LCA, left common carotid artery; LSA, left subclavian artery; LV, left ventricle; RA, right atrium; RV, right ventricle

A 31-year-old pregnant woman (gravida 1, para 0) was referred to our hospital for fetal echocardiography (Voluson E10; GE Healthcare, Chicago, IL, USA) at 25 weeks’ gestation. Echocardiographic examination revealed fetal PMV associated with coarctation of the aorta (CoA) (Fig. 1). In the four-chamber view, the mobility of the mitral valve leaflets was obviously limited without thickening and the shape of the mitral valve leaflets and some chordae was similar to “Ω” symbol in diastole (Fig. 1a and Movie). Further tracing demonstrated that all the chordae inserted into the posteromedial PM. The short-axis view of the left ventricle detected the posteromedial PM and did not identify the anterolateral PM (Fig. 1b). These findings led to a diagnosis of PMV. A systematic scan of the fetal heart revealed CoA and a small left ventricle (aortic isthmus, Z score − 3.47; left ventricular end-diastolic dimension, Z score − 2.41; left ventricular inlet length, Z score − 1.82). After a multidisciplinary consultation, including with a pediatric cardiac surgeon, the parents decided to terminate the pregnancy at 30 weeks’ gestation in view of the high surgical risks and difficulties associated with reoperation.

**Fig. 2:**
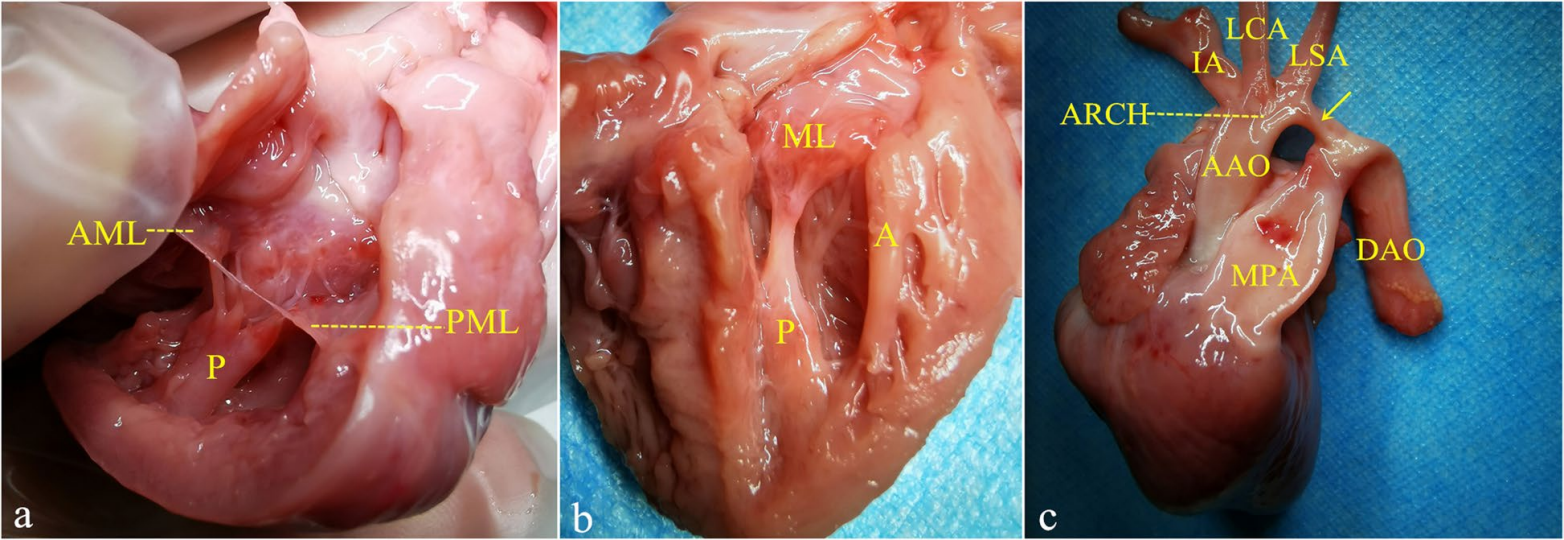
The fetal heart at autopsy. **a** All chordae inserted into the posteromedial PM with reduction of the interchordal spaces and diameter at the mitral valve opening. **b** The anterolateral PM was elongated and its lateral side is attached to the left ventricle wall without being connected by the chordae. **c** The aortic arch is narrowed, especially at the isthmus (arrow). A, anterolateral papillary muscle; AAO, ascending aorta; AML, anterior mitral valve leaflet; ARCH, aortic arch; DAO, descending aorta; IA, innominate artery; LCA, left common carotid artery; LSA, left subclavian artery; MPA, main pulmonary artery; P, posteromedial papillary muscle; PML, posterior mitral valve leaflet

Autopsy of the fetal heart and copy number variation sequencing were performed with the informed consent of the parents. Autopsy revealed that all chordae were connected to the elongated posteromedial PM (Fig. 2a) with narrowing of the interchordal spaces and a decreased diameter at the mitral valve opening. The anterolateral PM was attached to the ventricular wall for its entire length and its tip was located near the mitral valve annulus (Fig. 2b). CoA was confirmed at the same time (Fig. 2c). Therefore, a diagnosis of PLAMV with CoA was established. The copy number variation was normal.

## Discussion

PMV is a rare congenital malformation of the mitral valve that mainly involves the PMs. According to the underlying anatomy, two subtypes of PMV have been described [2, 3], namely, “true” PMV, which is characterized by a single PM that receives all chordae, and PLAMV, which has two PMs with unevenly distributed chordae. PLAMV is further divided into three grades according to the degree of uneven distribution of the chordae. The most common form is Grade II, in which a few short chordae are attached to the elongated PM [3]. Grade Ш is the most severe form [3]. The present case was found to have a grade Ш PLAMV with two PMs, one of which received all the chordae, while the other PM was attached by its lateral side to the wall of the left ventricle with the tip located near the mitral valve annulus. Disturbed delamination of the anterior or posterior part of the trabecular ridge from the left ventricular wall, combined with underdevelopment of chordae, seems to be the cause of PLAMV [4]. And “ture” PMV develops when the connection between the posterior and anterior part of the ridge condenses to form a single PM [4]. Although their anatomy and embryonic development are different, there is no obvious difference in the clinical manifestations, treatment, or prognosis between PLAMV and “true” PMV [2, 5]. Therefore, most clinical researchers have considered them to be one category.

PMV usually results in congenital MS and is often associated with multiple levels of left heart obstruction, known as Shone’s syndrome. There is limited information on fetal PMV in the literature, but there have been some isolated reports of MS as a manifestation of Shone’s syndrome [1, 6]. However, congenital MS is a morphologically heterogenous lesion that is classically divided into four anatomic types [7]: typical congenital MS, hypoplastic mitral valve, supramitral valve ring, and PMV. The different types of congenital MS require different treatment strategies and have different prognoses. Although the long-term functional outcome in children with congenital MS is satisfactory, surgical procedures for PMV are more complicated, and repeat repair may be needed [8, 9]. It is important to be able to identify the anatomic type of MS for prenatal counseling of parents with regard to treatment strategies and the prognosis.

Prenatal diagnosis of PMV by ultrasound is challenging, and the condition is usually diagnosed after birth. Fetal MS has often been described, but there are few reports on PMV [1, 6]. A restrictive opening of the mitral valve is an important clue for fetal MS. Further differential diagnosis of MS has great value in terms of prenatal consultation, but is difficult. In both children and adults, the echocardiographic characteristics of PMV include a single PM at the mid-papillary level and parachute leaflets at the basal level in the left ventricular short-axis view, doming of the elongated chords in diastole, and an enlarged left atrium in the four-chamber view [9, 10]. The short-axis view of the left ventricle and the four-chamber view are important when evaluating anomalies of the mitral valve. However, the anatomy of the PMs could not be accurately identified in the fetus [1]. In our case, fetal echocardiography failed to demonstrate the anterolateral PM either in the short-axis view of the left ventricle or in the four-chamber view. Therefore, diagnosis of PMV by observing the anatomy of the PMs is unreliable. However, we found that observing the morphology and movement of the valve leaflets and chordae, especially in the four-chamber view, was useful for prenatal diagnosis of PMV. In PMV, all the chordae are attached to one single PM with marked reduction in the interchordal spaces, which leads to specific limitation of movement of the leaflets, especially at the tips. On ultrasound images in the four-chamber view, parts of the leaflets and converged chordae are shaped like an arched bridge, and the overall mitral valve orifice is shaped like the symbol “Ω” in diastole, which is a key diagnostic clue for PMV. The mitral valve “Ω” sign can also be used to differentiate PMV from typical congenital MS and hypoplastic mitral valve. Ultrasonographic features of typical congenital MS is characterized by thickened and rolled leaflets, thickened and shortened chords, and restrictive opening of the mitral valve [11]. In a hypoplastic mitral valve, all components of the mitral valve are miniature versions of those of a normal valve [11].

## Conclusion

We have encountered a case of fetal PMV with CoA that was diagnosed by echocardiography and confirmed at autopsy. The “Ω” sign in a fetal mitral valve is an important clue for diagnosis of PMV. This sign provides a strong basis for the diagnosis of fetal PMV in the second trimester of pregnancy and may help to improve the ability to detect this entity.

## Supplementary Information

**Additional file 1.**

## Data Availability

The data and materials are available from the corresponding author on reasonable request.

## References

[1] Zucker N, Levitas A, Zalzstein E (2004). Prenatal diagnosis of Shone’s syndrome: parental counseling and clinical outcome. Ultrasound Obstet Gynecol.

[2] Marino BS, Kruge LE, Cho CJ (2009). Parachute mitral valve: morphologic descriptors, associated lesions, and outcomes after biventricular repair. J Thorac Cardiovasc Surg.

[3] Oosthoek PW, Wenink AC, Macedo AJ, Gittenberger-de Groot AC (1997). The parachute-like asymmetric mitral valve and its two papillary muscles. J Thorac Cardiov Sur.

[4] Oosthoek PW, Wenink AC, Wisse LJ (1998). Development of the papillary muscles of the mitral valve: morphogenetic background of parachute-like asymmetric mitral valves and other mitral valve anomalies. J Thorac Cardiovasc Surg.

[5] Delmo Walter EM, Javier M, Hetzer R. Repair of parachute and hammock valve in infants and children: early and late outcomes. Semin Thoracic Surg. 2016;448. 10.1053/j.semtcvs.2016.04.011. 10.1053/j.semtcvs.2016.04.011 28043459

[6] Weber RW, Ayala-Arnez R, Atiyah M (2013). Foetal echocardiographic assessment of borderline small left ventricles can predict the need for postnatal intervention. Cardiol Young.

[7] Ruckman RN, Van Praagh R (1978). Anatomic types of congenitall mitral stenosis: report of 49 autopsy cases with consideration of diagnosis and surgical implications. Am J Cardiol.

[8] Delmo Walter EM, Hetzer R (2018). Repair for congenital mitral valve stenosis. Semin Thorac Cardiovasc Surg Pediatr Card Surg Annu.

[9] Yuan S-M (2020). Parachute mitral valve: morphology and surgical management. Turk J Thorac Cardiovasc Surg.

[10] Hakim FA, Kendall CB, Alharthi M (2010). Parachute mitral valve in adults—a systematic overview. Echocardiography.

[11] Burbano NH (2020). Congenital Mitral Stenosis. J Cardiothorac Vasc Anesth.

