# Supplementary material for: Can prenatal diagnosis of parachute mitral valve be achieved? A case report of fetal parachute mitral valve
Source: Cardiovasc Ultrasound. 2022 Jul 8;20:16. doi: 10.1186/s12947-022-00288-z (PMC9264502; doi:10.1186/s12947-022-00288-z)
Supplement: Supplementary file 1 — Additional file 1. [file 12947_2022_288_MOESM1_ESM.zip › supplementary Movie legends.docx]

Movie. Fetal echocardiography in the four-chamber view showing thickened chordae that have converged into a single papillary muscle with narrowing of the interchordal spaces and limited movement of the mitral valve. In diastole, the anterior leaflet, posterior leaflet, and some chordae are shaped like the "Ω" symbol (arrow).

a, anterior mitral valve leaflet; LA, left atrium; LV, left ventricle; RA, right atrium; RV, right ventricle; p, posterior mitral valve leaflet
